# Transcriptional signatures of steroid hormones in the striatal neurons and astrocytes

**DOI:** 10.1186/s12868-017-0352-5

**Published:** 2017-04-05

**Authors:** Marcin Piechota, Michał Korostynski, Slawomir Golda, Joanna Ficek, Danuta Jantas, Ziolkowska Barbara, Ryszard Przewlocki

**Affiliations:** 1 Department of Molecular Neuropharmacology, Institute of Pharmacology Polish Academy of Sciences, Smetna 12, 31-343 Kraków, Poland; 2 Department of Neuroendocrinology, Institute of Pharmacology Polish Academy of Sciences, Smetna 12, 31-343 Kraków, Poland

## Abstract

**Background:**

The mechanisms of steroids actions in the brain mainly involve the binding and nuclear translocation of specific cytoplasmic receptors. These receptors can act as transcription factors and regulate gene expression. However, steroid-dependent transcriptional regulation in different types of neural cells is not yet fully understood. The aim of this study was to evaluate and compare transcriptional alterations induced by various steroid receptor agonists in primary cultures of astrocytes and neurons from mouse brain.

**Results:**

We utilized whole-genome microarrays (Illumina Mouse WG-6) and quantitative PCR analyses to measure mRNA abundance levels. To stimulate gene expression we treated neuronal and astroglial cultures with dexamethasone (100 nM), aldosterone (200 nM), progesterone (200 nM), 5α-dihydrotestosterone (200 nM) and β-Estradiol (200 nM) for 4 h. Neurons were found to exhibit higher levels of expression of mineralocorticoid receptor, progesterone receptor and estrogen receptor 2 than astrocytes. However, higher mRNA level of glucocorticoid receptor mRNA was observed in astrocytes. We identified 956 genes regulated by steroids. In astrocytes we found 381 genes altered by dexamethasone and 19 altered by aldosterone. Functional classification of the regulated genes indicated their putative involvement in multiple aspects of cell metabolism (up-regulated *Slc2a1*, *Pdk4* and *Slc45a3*) and the inflammatory response (down-regulated *Ccl3*, *Il1b* and *Tnf*). Progesterone, dihydrotestosterone and estradiol did not change gene expression in astrocytes. We found no significant changes in gene expression in neurons.

**Conclusions:**

The obtained results indicate that glial cells might be the primary targets of transcriptional action of steroids in the central nervous system. Substantial changes in gene expression driven by the glucocorticoid receptor imply an important role for the hypothalamic–pituitary–adrenal axis in the hormone-dependent regulation of brain physiology. This is an in vitro study. Hence, the model may not accurately reflect all the effects of steroids on gene expression in neurons in vivo.

**Electronic supplementary material:**

The online version of this article (doi:10.1186/s12868-017-0352-5) contains supplementary material, which is available to authorized users.

## Background

The crucial components of the neuroendocrine system are steroid hormones. They affect the central nervous system by regulating allostasis and by coordinating the responses to external and internal stimuli [1]. The most prominent example of steroid impact on the brain is the release of glucocorticoids after stressful stimulus [2]. Elevated levels of these adrenal steroids may subsequently affect sleep [3], cognition [4], memory [5] or mood [6]. Moreover, our previous research revealed that glucocorticoids mediate the effects on gene expression in the striatum induced by multiple psychoactive drugs, including antidepressants (fluoxetine, mianserin), antipsychotics (risperidone, clozapine) and drugs of abuse (heroin, ethanol) [7, 8]. This is probably caused by both direct and indirect actions of psychoactive drugs on the hypothalamus and later on the pituitary and adrenal glands [8]. Furthermore, the association between dysregulation of the hypothalamic–pituitary–adrenal (HPA) axis and major depression is among the most consistent biological findings in psychiatry [9]. The other steroids including mineralocorticoids, androgens, estrogens and progestogens also influence the central nervous system [10, 11].

Steroids bind to nuclear steroid receptors, which then bind to their response elements on DNA, activating gene transcription and causing changes in gene expression. The alterations in transcript abundance mediated by these receptors are diverse and vary among cell types. It has been shown that the cell-type-specific profile of glucocorticoid receptor occupancy is predetermined by differences in the chromatin accessibility pattern [12]. Moreover, various cell populations differ in levels of steroid receptor genes expression. The most remarkable difference between various cell types in the central nervous system is high expression of progesterone receptor (*Pgr*) in neurons in contrast to glial and epithelial cells [13]. This cellular specificity contrasts with the lower specificity of steroid receptor ligands and DNA response elements. For example, corticosterone—an adrenal steroid—binds to both mineralocorticoid and glucocorticoid receptors [14]. Furthermore, the differences in androgen and glucocorticoid receptor DNA binding properties are quantitative rather than qualitative [15].

The crucial aspect of steroids action on the central nervous system is how these hormones affect various cellular populations involved in control of behaviour. It is particularly interesting in the striatum, a brain region that is responsible for steroid-related neuropsychiatric phenotypes including mood control [16] and reward processing [17]. To date, there have been few studies comparing various neural cell-type-specific responses to steroids. Slezak et al. [18] compared the responses of astrocytes and neurons to dexamethasone. Jenkins et al. [19] compared the responses of oligodendrocytes, astrocytes and microglia to the same steroid. The transcriptional responses of these cell types to dexamethasone were diverse. Therefore, we aimed to infer the role of various steroids in the function of astrocytes and neurons, based on gene expression changes caused by these substances. Here, we present gene expression profiling of primary neuronal and astroglial cultures treated with various steroid receptor agonists: dexamethasone, aldosterone, progesterone, estradiol and dihydrotestosterone.

## Methods

### Animals

The animals used in the study were from the C57BL/6 J inbred strain colony that is maintained at the animal facility of the Institute of Pharmacology of the Polish Academy of Sciences in Krakow, Poland. The mice were housed 6–10 per cage, under a 12-h dark/light cycle, with free access to food and water. The mice had not been subjected to any prior testing. The experimental protocols used in the study were approved by the local Bioethics Commission at the Institute of Pharmacology, Polish Academy of Sciences (Krakow, Poland).

### Primary cultures of striatal neurons and astrocytes

**Fig. 1:**
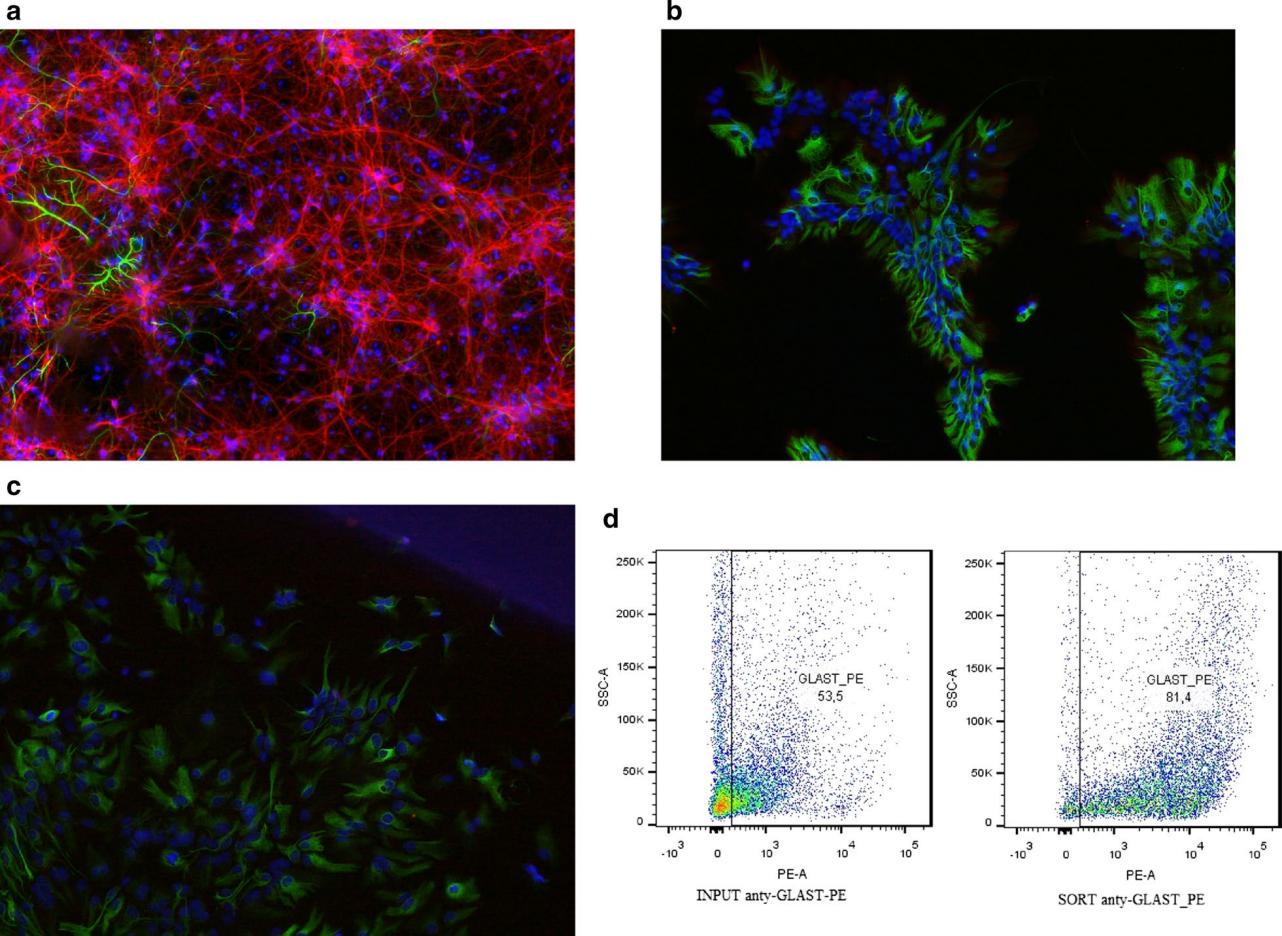
Primary cell cultures of neurons and astrocytes. The purity was estimated by immunostaining for specific protein markers. (**a**) Neuronal cultures were immunostained on glass slides against MAP2 protein (*red*), GFAP astrocyte specific protein (*green*) and DAPI (*blue*). The staining confirmed at least 90% purity of neuronal cultures from the mouse striatum. (**b**) Primary astrocytes stained against GFAP (*green*), MAP2 (*red*) and DAPI (*blue*). (**c**) Primary astrocytes stained against GFAP (*green*), NEUN (*red*) and DAPI (*blue*). (**d**) The enrichment of astrocytes was determined by flow cytometry of immunostained GLAST-positive cells isolated from the brains of 5 days old C57BL/6 J mice. Approximately 80% of the cells were GLAST-positive astrocytes after the separation on MACS columns

The ex vivo experiments were conducted using mouse striatal neurons and astrocytes from primary cultures. Neuronal tissues were dissected from mouse embryos at 17–18 days of gestation. The tissues were cut into small pieces and digested with trypsin (0.1% for 15 min at RT). The cells were suspended in Neurobasal^®^ medium (Gibco) and plated onto polyornithine multiwell plates (TPP). After 2 days, the culture medium was replaced with Neurobasal medium supplemented with B27 (50 μl/100 ml). This procedure typically yields cultures that contain >90% neurons (Fig. 1). Primary astrocytes were isolated from P5 to P7 mice. The brains were collected, dissected and digested using the Neural Tissue Dissociation Kit (T) (Miltenyi Biotec GmbH, Bergisch Gladbach, Germany) according to the manufacturer’s protocol. Astrocytes from single-cell suspensions were isolated using the Anti-GLAST (ACSA-1) MicroBead Kit (Miltenyi Biotec), separated on MACS MS Columns using the MACS magnetic separator, seeded on 24-well plates and grown in high-glucose DMEM supplemented with 10% FBS and antibiotics for 7 days. All cell cultures were maintained at 37 °C in a humidified atmosphere containing 5% CO_2_ for 7 days prior to experiments. Both astrocytes and neurons were cultured in Neurobasal medium with no supplementation for the final 24 h.

### Steroid ligands ex vivo treatments

Both neurons and astrocytes were exposed to dexamethasone (100 nM), corticosterone (10, 50 and 200 nM) aldosterone (200 nM), progesterone (200 nM), 5α-dihydrotestosterone (200 nM), or β-Estradiol (200 nM) for 4 h and 8 h (corticosterone only). The doses were selected based on previous studies [20–22]. To obtain stock solutions 1 mg of each compound was was dissolved in 100% ethanol and then diluted 50 times in neurobasal medium without supplements. As a result of dissolution we obtained stock solutions of dexamethasone (51 μM), corticosterone (57 μM), ß-estradiol (73 μM), progesterone (64 μM), aldosterone (55 μM) and testosterone (69 μM). The highest final solution of ethanol in culture medium was below 0.004% and this concentration was used as the vehicle negative control. The 4 h time point was selected based on previous in vitro studies using dexamethasone [18] and in vivo studies using progesterone and glucocorticoid receptor antagonist RU486 [8].

### RNA isolation

RNA was isolated following the manufacturer’s protocol and was further purified using the RNeasy Mini Kit (Qiagen Inc.). Total RNA concentration was measured using a NanoDrop ND-1000 (NanoDrop Technologies Inc., Montchanin, DE, USA). RNA quality was determined using an Agilent 2100 Bioanalyzer (RIN > 8)(Agilent, Palo Alto, CA, USA).

### Quantitative PCR

Reverse transcription was performed using Omniscript Reverse Transcriptase (Qiagen Inc.). TaqMan^®^ probes (Life Technologies) were used for the qPCR assays, which were run on the CFX96 Real-Time system (BioRad). Each template was generated from an individual sample, and the amplification efficiency for each assay was determined by running a standard dilution curve. Expression of the hypoxanthine–guanine phosphoribosyltransferase 1 (*Hprt1*) transcript, which showed stable levels following the treatment was quantified to control for variation in cDNA amounts. The abundance of RNA was calculated as 2^−(threshold cycle)^. Data were analyzed using Student’s *t* test.

### Gene expression profiling

First, 200 ng high-quality total RNA was used to generate cDNA and cRNA with the Illumina TotalPrep RNA Amplification Kit (Illumina Inc., San Diego, CA, USA). The procedure consisted of reverse transcription with an oligo(dT) primer bearing a T7 promoter using an Array-Script. The obtained cDNA became a template for in vitro transcription using T7 RNA polymerase and biotin UTP, which generated multiple copies of biotinylated cRNA. The purity and concentration of the cRNA were checked using an ND-1000 Spectrometer. Quality cRNA was then hybridized using Illumina’s direct hybridization array kit (Illumina). Each cRNA sample (1.5 μg) was hybridized overnight to the MouseWG-6 BeadChip arrays (Illumina) in a multiple-step procedure according to the manufacturer’s instructions; the chips were washed, dried and scanned on the BeadArray Reader (Illumina). Raw microarray data were generated using BeadStudio v3.0 (Illumina). Four biological replicates of the microarrays were prepared per experimental group. A total of 48 Illumina MouseWG-6 v2 microarrays (with probes for approximately 48,000 transcripts) were used in the experiment. To provide an appropriate balance in the whole dataset, groups were equally divided between the array hybridization batches.

### Microarray data analysis

Microarray quality control was performed using BeadArray R package. The following parameters were checked: number of outliers, number of beads and percentage of probes detected. After background subtraction, the data were normalized using quantile normalization and then log_2_-transformed. The obtained signal was taken as the measure of mRNA abundance derived from the level of gene expression. Statistical analysis of the results was performed using two-way ANOVA (for the factors drug and cell type) followed by false discovery rate (FDR) correction for multiple testing. To obtain drug-versus-control comparisons, a t-test was used followed by FDR correction. All statistical analyses were performed in R software.

### Cluster analysis

Hierarchical clustering was performed using the measure of correlation and complete linkage method. The cluster separation was performed based on up- and downregulation of transcripts. The functional annotation analysis tool DAVID was used to identify over-represented ontologic groups among the gene expression patterns and to group genes into functional classifications. For cell-type enrichment of mRNA, a recently published brain transcriptome database was used [13]. The identification of over-represented transcription factor binding sites was performed using the seqinspector database with default parameters [23].

## Results

### Differences in transcripts levels of steroid receptors between astroglial and neuronal primary cultures

**Fig. 2:**
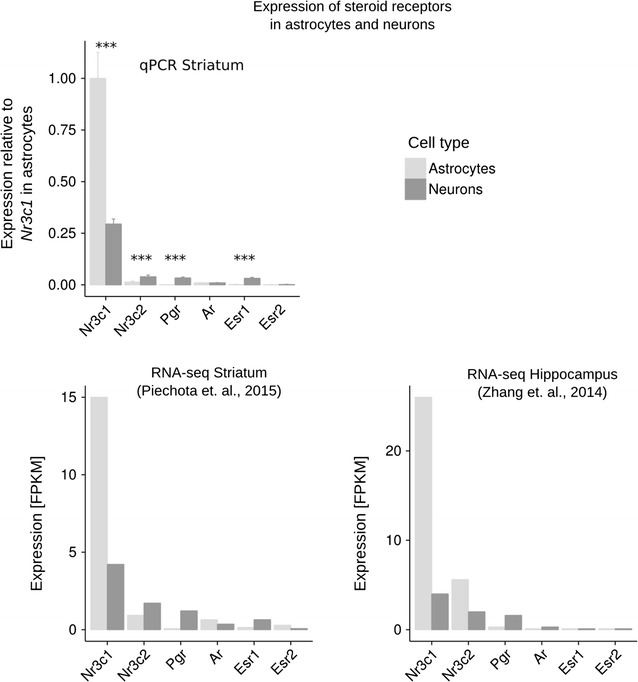
Levels of steroid hormone receptors in astroglial and neuronal primary cultures. Bar graphs (*upper left*) summarizing the qPCR-based measurement of gene expression of steroid nuclear receptors *Nr3c1, Nr3c2, Pgr, Ar, Esr1, Esr2* (n = 4–6). Significant differences in the expression between astrocytes and neurons are indicated by asterisks (*t* test, ***p < 0.001). Bar graphs (*bottom left*) summarizing RNA-seq-based measurement of gene expression of steroid nuclear receptors [24]. Bar graphs summarizing RNA-seq-based measurement of gene expression of steroid nuclear receptors as reported by [13]. Technique and tissue from which the primary cultures were derived are indicated in the titles of the particular plots

We estimated levels of nuclear steroid receptors in neuronal and astroglial primary cultures using quantitative PCR (Fig. 2; upper panel). There was no difference between neurons and astrocytes in the expression of androgen receptor (*Ar*) and estrogen receptor 2 (*Esr2*) (*p* > 0.05). Neurons showed higher expression of mineralocorticoid receptor (*Nr3c2*) (*p* = 0.0005), progesterone receptor (*Pgr*) (*p* = 3.6 × 10^−7^), and estrogen receptor 1 (*Esr1*) (*p* = 1.1 × 10^−6^). Astrocytes showed higher levels of abundance of glucocorticoid receptor (*Nr3c1*) (*p* = 7.4 × 10^−7^). In addition, to compare mRNA levels between the receptors we used the available RNA-seq data from previous experiments by Piechota et al. [24] (Fig. 2; middle panel) and by Zhang et al. [13] (Fig. 2; lower panel). Consistent with the qPCR results, the previously published RNA-Seq studies by Piechota et al. [24] showed higher levels of expression of *Nr3c2*, *Pgr* and *Esr1* in neurons and higher levels of expression of *Nr3c1* in astrocytes. RNA-seq data published by Zhang et al. [13] were similar to qPCR results with two differences—astrocytes exhibited higher levels of *Nr3c2*, and mRNA of *Esr1* was undetected.

### Transcriptome alterations induced by steroid receptors agonists

We treated neuronal and astroglial primary cultures with dexamethasone (100 nM), aldosterone (200 nM), progesterone (200 nM) and estradiol (200 nM) for 4 h. For gene expression profiling, we used Illumina Mouse WG-6 v2 microarrays. By performing two-way ANOVA (with the factor cell type and drug) we identified 956 genes regulated by steroids (FDR < 1%). In astrocytes we found 381 genes altered by dexamethasone (*t* test, FDR 1%), 19 by aldosterone, 0 by progesterone (9 at FDR 20% threshold), and 0 by dihydrotestosterone and estradiol. In neurons we identified 0 genes regulated by dexamethasone (19 at FDR 20%), and 0 by aldosterone, progesterone, dihydrotestosterone and estradiol. We performed gene ontology analysis of genes regulated by dexamethasone (n = 381), aldosterone (n = 19) and progesterone (n = 9 at less restrictive threshold). The complete lists of regulated genes are presented in the supplementary materials (Additional file 1).

We performed functional analyses of genes regulated by dexamethasone, aldosterone and progesterone. Using the DAVID tool we looked for overrepresented gene ontology terms and molecular pathways. We found that genes upregulated by dexamethasone are functionally heterogeneous and include only a few overrepresented terms: positive regulation of cell proliferation (*p* = 1.0 × 10^−5^; FDR = 0.014; e.g. *Bcl2l1*, *Cdkn1a*, *Trp63*), negative regulation of inflammatory response (*p* = 0.0015; FDR = 0.21; *Ada*, *Zfp36*), cell junction (*p* = 0.012; FDR = 0.36; *Amica1*, *Gjb6*). The genes downregulated by dexamethasone are functionally related to the immune response: toll-like receptor signaling pathway (*p* = 5.7 × 10^−8^; FDR = 4.1 × 10^−6^; *Ccl3*, *Il1b*, *Tnf*), cytokine–cytokine receptor interaction (*p* = 5.2 × 10^−5^; FDR = 1.9 × 10^−3^; *Ccl12*, *Cxcr10*), or immune response (*p* = 1.2 × 10^−4^; FDR = 0.098; *Tlr2*, *Igh*-6). We did not find any overrepresented terms for genes regulated by aldosterone or progesterone.

We evaluated transcriptional mechanisms controlling gene expression using the seqinspector tool [23] that utilize previously published ChIP-seq experiments [12, 25]. Promoters of genes upregulated by dexamethasone in astrocytes were enriched in ChIP-seq signal for glucocorticoid receptor (SRA id: SRA027300, *p* = 16 × 10^−10^). Promoters of genes downregulated by dexamethasone in astrocytes have binding sites for E2F1 (GEO id: GSM881059, *p* = 5.2 × 10^−8^), REL (GEO id: GSM881134, *p* = 4.7 × 10^−6^), RELA (GEO id: GSM881114, *p* = 6.0 × 10^−6^), JUNB (GEO id: GSM881079, *p* = 6.3 × 10^−6^), STAT2 (GEO id: GSM881162, *p* = 5.3 × 10^−5^), IRF4 (GEO id: GSM881150, *p* = 5.9 × 10^−5^), NFKB1 (GEO id: GSM881154, *p* = 1.2 × 10^−4^). Promoters of genes upregulated by aldosterone in astrocytes contain binding sites for glucocorticoid receptor (SRA id: SRA027300, *p* = 1.1 × 10^−14^). Genes regulated by progesterone in astrocytes have binding sites for BHLHE40 (GEO id: GSM912922, *p* = 2.3 × 10^−9^) and glucocorticoid receptor (SRA id: SRA027300, *p* = 8.7 × 10^−9^).

### Gene expression changes induced by corticosterone

**Fig. 3:**
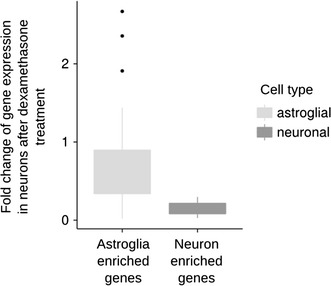
Dexamethasone-induced regulation of astroglia specific genes in neuronal primary cultures. The *bar graph* summarizes the induction of gene expression in neurons after dexamethasone treatment. Two groups of genes are presented: astrocyte-specific genes (log_2_ ratio of astroglial to neuronal expression higher than 2) and neuron-specific genes (log_2_ ratio of astroglial to neuronal expression lower than −2)

We compared transcriptional alterations induced by dexamethasone and aldosterone to endogenous non-specific glucocorticoid and mineralocorticoid receptors agonist, corticosterone. The effects of corticosterone on gene expression were measured in neurons and astrocytes in time and dose dependent manner by using quantitative PCR. From the list of genes detected by microarray profiling we selected three previously known glucocorticoid-receptor-dependent transcripts (*Fkbp5, Zbtb16* and *Sgk1*) (Fig. 3). For statistical analysis we used three way ANOVA for cell type, dose and time factors. Interaction between factors was statistically significant (*p* < 0.05) for all of the transcripts examined. Tukey HSD test was used as a post hoc test. All inspected transcripts were significantly upregulated in astrocytes (at doses of 50 nM and 200 nM) in both 4 h and 8 h time points. None of the inspected transcripts were significantly regulated in neuronal cells.

### Glial enrichment of dexamethasone induced gene expression alterations in neurons

**Fig. 4:**
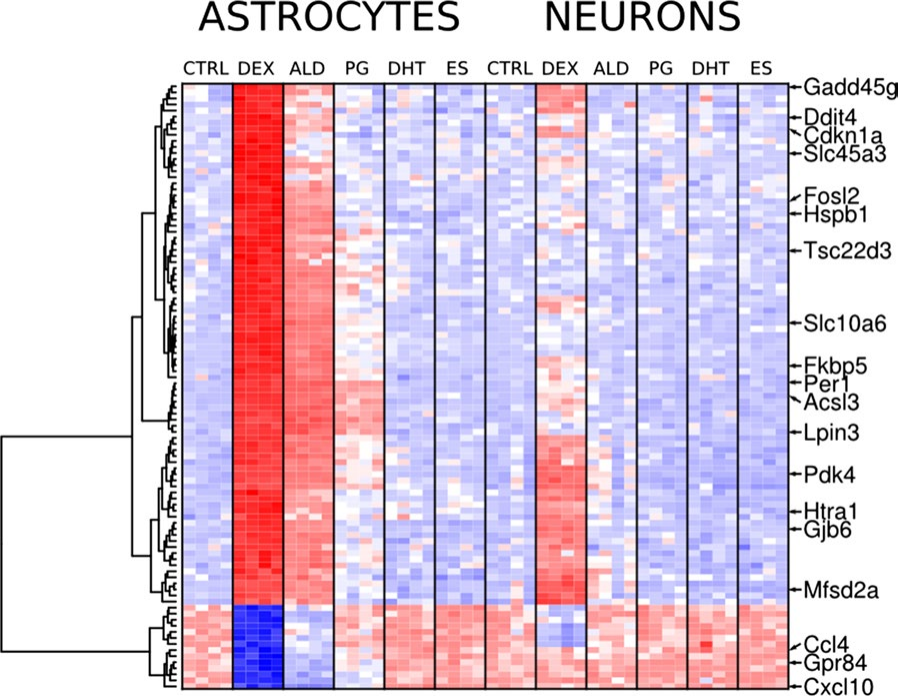
Steroid receptor agonist induced transcriptome alterations. Microarray results are shown as a heat map and include the top 100 genes with significant differences shown by two-way ANOVA for the drug factor. Colored rectangles represent transcript abundance in neurons and astrocytes in control samples (CTRL) and after treatment with dexamethasone (DEX), aldosterone (ALD), progesterone (PG), dihydrotestosterone (DHT) or estradiol (ES). The intensity of the color is proportional to the standardized values (*blue* low, *red* high). Clustering was performed using correlation distance and complete tree-building method. Particularly interesting gene symbols are shown on the right. The data for neurons and astrocytes were adjusted for means in the control groups to better visualise the effects of steroid compounds. FPKM—fragments per kilobase of exon per million fragments mapped

**Fig. 5:**
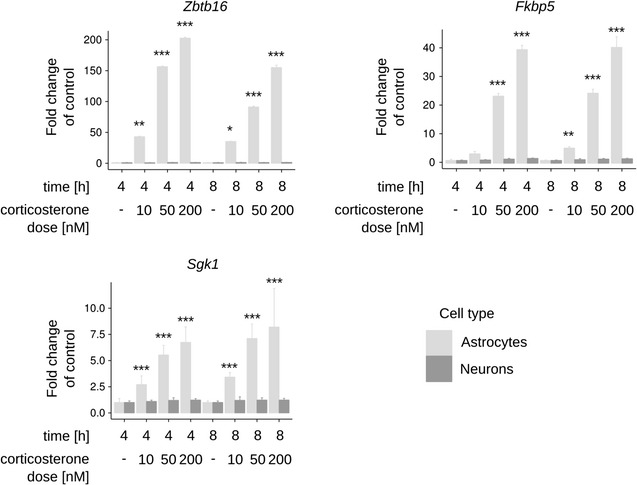
Effects of corticosterone on gene expression of selected genes in astrocytes and neurons. *Bar graphs* summarizing the qPCR-based measurements of changes in the expression of selected genes following the indicated corticosterone dose and time. Data are presented as the fold change over the control group (without corticosterone) ± standard deviation (n = 3–4). Significant differences from the post hoc analysis using a Tukey’s honest significant difference test (vs. appropriate control) by *asterisks* (***p < 0.001, **p < 0.01 and *p < 0.05)

We observed that the genes induced in neurons (top 20, *t* test FDR < 26%) were expressed at higher levels in astrocytes (*p* = 0.0017, *t* test between expression levels for controls in neurons and astrocytes). Using the top 100 genes identified by two-way ANOVA in the whole experiment and compared fold induction of astroglia and neuron-specific genes in dexamethasone treated neurons (Fig. 4). In neuronal primary cultures, we observed higher induction of dexamethasone-induced astroglia enriched genes than dexamethasone-induced neuron enriched genes (*p* = 0.032, *t* test between fold changes in gene expression) (Fig. 5).

## Discussion

The most remarkable observation of this study was the weak, or absent response of neurons to hormonal stimulus. As has previously been suggested, the main component of transcriptional response to adrenal steroids is not neuronal but astroglial [8, 26, 27]. This hypothesis was based on astrocyte-specific gene enrichment (higher level of expression) among glucocorticoid inducible transcripts [26]. In our previous studies, we have also shown that an inducible form of *Sgk1* is present in astrocytes and not in neurons [18]. In that study, we found that, unlike astrocytes, neurons are not transcriptionally responsive to dexamethasone treatment. We hypothesized that in the previous experiments, the lack of transcriptional response of neurons to dexamethasone might have been caused by cell culture conditions and low concentration of dexamethasone (10 nM). Thus, in this study neurons and astrocytes were cultured in the same manner for the final 3 days. However, despite all those previous indications, we were startled by the low induction of gene expression in neurons. The most unexpected result was the lack of response of neurons to progesterone, despite the high expression of progesterone receptor [13] and the promotion of neuronal survival by this steroid [28, 29]. The presented in vitro study has several limitations. The model may not accurately reflect the effect of steroids on gene expression in neurons in the brain. It is possible that neurons isolated from embryonic tissue are subsensitive to steroids. Such a mechanism may insulate the brain from developmental interference from external steroids such as xenobiotic or maternally-generated steroids. Neonatal neurons cultured for 7 days are not fully developed and may have a lower number of functional synapses compared to mature cells. Moreover, our study was designed to detect changes with twofold or higher, with a power of >50%. Thus, smaller changes in mRNA abundance levels in neurons might have gone undetected.

Neither neurons nor astrocytes responded to estradiol or dihydrotestosterone, likely because of low levels of these receptors in our cultures. Although these steroids have not acted transcriptionally in our case, they might act on membrane channels and receptors such as GABAA [30]. Although the astrocytes express far fewer progesterone receptors [13] than do neurons, they responded to progesterone transcriptionally. Nevertheless, this response was much weaker than those caused by dexamethasone or aldosterone. Astrocytic response to dexamethasone and aldosterone were qualitatively similar. This pattern of transcriptional activation was also detected in neuronal cells. However, the profile of changes indicates the very small number of contaminating astrocytes as a source of gene expression alterations. These results suggest that in our experiment response of neuronal cells to steroid hormones is repressed.

The mechanism of steroid nuclear receptor repression in neurons is intriguing. Our first hypothesis was focused on non-active isoforms of these receptors. We verified this speculation by reviewing the RNA-seq brain gene expression atlas [13] and FANTOM5 cage data [31]. In fact, the transcriptional isoform of *Pgr* expressed in neurons is shorter and has not been previously annotated in the Ensembl database. However, neurons and astrocytes express the same transcriptional isoforms of *Nr3c1* and *Nr3c2*, which disproves this conjecture. Our second hypothesis concerned the absence of nuclear receptor binding sites in neurons. The chromatin availability predetermines the binding sites of glucocorticoid receptor [12]. Nonetheless, such a complete blockade is rather unlikely. Our third hypothesis involves an imbalance between coactivators and corepressors of steroid receptors in neurons which would inhibit their transcriptional activity [32–34] and would allow only nongenomic mechanisms of action [35, 36] (see expanded discussion in Additional file 2).

The genes regulated in astrocytes in vitro by dexamethasone overlap with other studies of transcriptional alterations induced by steroids in vivo. Nine out of the top ten genes regulated by dexamethasone in mouse hypothalamus are also regulated in primary astrocytes (i.e. *Gadd45* *g, Sgk1, Pdk4, Nfkbia, Plekhf1, Errfi1, Arrdc2, Dusp1* and *Bcl6*) [37]. Similarly, multiple genes regulated by corticosterone in the rat hippocampus were identified in the presented study (e.g. *Bcl6, Fkbp5, Gjb6, Errfi1, Pdk4*) [38]. The genes upregulated by dexamethasone and aldosterone in astrocytes are functionally heterogeneous, which suggests diverse molecular mechanisms of action. One of these mechanisms may be related to the regulation of energy supply to neuronal cells. Genes involved in this process include a major glucose transporter in the mammalian blood–brain barrier *Slc2a1* [39], which was upregulated after dexamethasone or aldosterone treatment. Another gene *Pdk4* maintains pyruvate dehydrogenase in the phosphorylated, inactive state and shunts pyruvate to form lactate in astrocytes [13]. Dexamethasone also upregulates the putative sugar transporter *Slc45a3* [40]. Another set of regulated genes represents control of a negative feedback on the action of steroids. The genes involved in this process include *Fkbp5* [41] and *Hspa1b* [42]. Astrocytes also show upregulation of the cell adhesion molecules *Amica1* and *Gjb6*, which might influence the extracellular matrix and support neuronal plasticity. The genes downregulated by dexamethasone are homogeneous and are involved in inflammatory response. Dexamethasone can effectively attenuate the inflammatory response to wounding, reducing neuronal and glial scarring in the vicinity of the wound [43].

## Conclusions

Our study sheds new light on the mechanisms of action of steroid hormones in the central nervous system. The results show that glial cells constitute a primary target for the rapid transcriptional action of steroids. Identification of genes that mediate effects of adrenal steroids on astrocytes revealed novel molecular mechanisms involved in regulation of brain metabolism and plasticity. The unexpectedly low transcriptional response of neuronal cells to steroids provides rationale for further in vivo studies on this issue.

## Additional files

**Additional file 1.** Lists of regulated genes. The complete list of genes regulated by dexamethasone, aldosterone, progesterone, dihydrotestosterone and estradiol. The list includes *p* values, corrected *p* values and log2 ratios for all pairwise comparisons.

**Additional file 2.** Cell expression analysis of glucocorticoid receptor cofactors.

## Abbreviations

P5: postnatal day 5
P7: postnatal day 7
FDR: false discovery rate
SRA: short read archive
GEO: Gene Expression Omnibus
Tukey HSD: Tukey’s honest significant difference test
CTRL: control
DEX: dexamethasone
ALD: aldosterone
PG: progesterone
DHT: dihydrotestosterone
ES: estradiol
FPKM: fragments per kilobase of exon per million fragments mapped
