# Supplementary material for: Transcriptional signatures of steroid hormones in the striatal neurons and astrocytes
Source: BMC Neurosci. 2017 Apr 5;18:37. doi: 10.1186/s12868-017-0352-5 (PMC5381047; doi:10.1186/s12868-017-0352-5)
Supplement: Supplementary file 2 — Additional file 2. Cell expression analysis of glucocorticoid receptor cofactors. [file 12868_2017_352_MOESM2_ESM.pdf]

Cell expression analysis of glucocorticoid receptor cofactors

We have prepared a list of 76 cofactors and coactivators of the steroid receptors. The expression levels of the selected genes were analyzed in neurons and astrocytes. We found two clusters of transcripts, the first group enriched in neurons, the second one in glia cells. As expected, both the groups contain genes involved in intracellular receptor signaling pathway (GO:0030522). Furthermore, functional analysis of the selected genes indicated interesting differences. We compared top 10 categories of GO Biological Processes over-represented (adjusted $p < 0.001$) in each cluster. Coactivators expressed at higher level in astrocytes are involved in development of embryonic placenta (GO:0001892) and regulation of transcription from RNA polymerase II promoter (GO:0006366). Coactivators expressedat a higher level in neurons are related to nucleosome disassembly (GO:0006337) and regulation of histone acetylation (GO:0035065). The observed differences may suggest that intracellular GR system is involved in active regulation of transcriptional processes in astrocytes and epigenomic modifications in neuronal cells.

List of glucocorticoid receptor cofactors and coactivators as defined by BioGrid 3.4:
Ada, Bag1, Ccdc6, Cd3g, Cd4, Cebpa, Cebpb, Crebbp, Dap3, Daxx, Ddx54, Egfr, Ep300, Fkbp4, Fkbp5, Fyn, Grip1, Hdac1, Hdac6, Hmox2, Hnrnpu, Hsp90aa1, Hspa1a, Kat5, Med1, Med25, Ncoa1, Ncoa2, Ncoa4, Ncoa6, Ncor1, Nedd4l, Nfkbia, Nr1h3, Nr2f2, Nr2f6, Nr3c1, Nr3c2, Nr4a1, Nrip1, Osgep, Pias2, Pou1f1, Pou2f1, Pou2f2, Prkdc, Psmd7, Ptges3, Ptms, Ranbp9, Rela, Rif1, Rps6ka5, Set, Sf3a1, Sfpq, Sgta, Smad3, Smarca4, Smarcd1, Stat5b, Stub1, Sumo1, Svil, Tada2l, Tbp, Tdg, Tgfb1i1, Trim24, Trim28, Trp53, Tsg101, Ttc5, Txn1, Txn2, Zbtb16.

Chatr-Aryamontri A, Breitkreutz BJ, Oughtred R, Boucher L, Heinicke S, Chen D, Stark C, Breitkreutz A, Kolas N, O'Donnell L, Reguly T, Nixon J, Ramage L, Winter A, Sellam A, Chang C, Hirschman J, Theesfeld C, Rust J, Livstone MS, Dolinski K, Tyers M. The BioGRID interaction database: 2015 update. Nucleic Acids Research. Nov. 2014
